# Evaluation of the effectiveness and cost-effectiveness of Families for Health V2 for the treatment of childhood obesity: study protocol for a randomized controlled trial

**DOI:** 10.1186/1745-6215-14-81

**Published:** 2013-03-20

**Authors:** Wendy Robertson, Sarah Stewart-Brown, Nigel Stallard, Stavros Petrou, Frances Griffiths, Margaret Thorogood, Douglas Simkiss, Rebecca Lang, Kate Reddington, Fran Poole, Gloria Rye, Kamran A Khan, Thomas Hamborg, Joanna Kirby

**Affiliations:** 1 Warwick Medical School, University of Warwick, Coventry, CV4 7AL, UK; 2 Public Health, NHS Coventry, Civic Centre 1, Little Park Street, Coventry, CV1 5RB, UK; 3 Public Health Department, NHS Warwickshire, PO Box 43, Shire Hall, Barrack Street, Warwick, CV34 4SX, UK; 4 Wolverhampton City Council, Civic Centre, St Peter’s Square, Wolverhampton, WV1 1RT, UK

**Keywords:** Childhood obesity, Weight management, Parenting, Randomized controlled trial, Economic evaluation

## Abstract

**Background:**

Effective programs to help children manage their weight are required. Families for Health focuses on a parenting approach, designed to help parents develop their parenting skills to support lifestyle change within the family. Families for Health V1 showed sustained reductions in overweight after 2 years in a pilot evaluation, but lacks a randomized controlled trial (RCT) evidence base.

**Methods/design:**

This is a multi-center, investigator-blind RCT, with parallel economic evaluation, with a 12-month follow-up. The trial will recruit 120 families with at least one child aged 6 to 11 years who is overweight (≥91st centile BMI) or obese (≥98th centile BMI) from three localities and assigned randomly to Families for Health V2 (60 families) or the usual care control (60 families) groups. Randomization will be stratified by locality (Coventry, Warwickshire, Wolverhampton).

Families for Health V2 is a family-based intervention run in a community venue. Parents/carers and children attend parallel groups for 2.5 hours weekly for 10 weeks. The usual care arm will be the usual support provided within each NHS locality.

A mixed-methods evaluation will be carried out. Child and parent participants will be assessed at home visits at baseline, 3-month (post-treatment) and 12-month follow-up. The primary outcome measure is the change in the children’s BMI *z*-scores at 12 months from the baseline. Secondary outcome measures include changes in the children’s waist circumference, percentage body fat, physical activity, fruit/vegetable consumption and quality of life. The parents’ BMI and mental well-being, family eating/activity, parent–child relationships and parenting style will also be assessed.

Economic components will encompass the measurement and valuation of service utilization, including the costs of running Families for Health and usual care, and the EuroQol EQ-5D health outcomes. Cost-effectiveness will be expressed in terms of incremental cost per quality-adjusted life year gained. A *de novo* decision-analytic model will estimate the lifetime cost-effectiveness of the Families for Health program.

Process evaluation will document recruitment, attendance and drop-out rates, and the fidelity of Families for Health delivery. Interviews with up to 24 parents and children from each arm will investigate perceptions and changes made.

**Discussion:**

This paper describes our protocol to assess the effectiveness and cost-effectiveness of a parenting approach for managing childhood obesity and presents challenges to implementation.

**Trial registration:**

Current Controlled Trials http://ISRCTN45032201

## Background

The prevalence of obesity in children aged 2 to 15 in England rose from 11.7% in 1995 to 18.9% in 2004 and, although the trend may now be flattening (16% in 2010), it remains high with a third of children classified as either overweight or obese [1]. There are an estimated 750,000 children aged 2 to 10 in England who are obese [2]. Obesity in childhood increases the risk of poor physical health in childhood, including type-2 diabetes and cardiovascular risk factors, particularly when obesity is severe [3]. Psychological well-being is impaired with low self-esteem and depression in children attending for treatment of obesity [4]. There is evidence that childhood obesity also affects adult health. Longitudinal studies indicate that a higher BMI in childhood or adolescence increases morbidity and mortality from coronary heart disease in adulthood [5,6] and is associated with adverse socio-economic outcomes in women [7,8]. Furthermore, 40% to 70% of obese children become obese adults [9], with the associated risks to adult health.

The prevention and management of childhood obesity is now a public health priority. Effective interventions are needed to treat children who are obese, in order to reduce ill-health in children and the proportion whose obesity continues into adulthood. A Cochrane systematic review of interventions to treat obesity identified 64 RCTs, and of these only two were from the UK [10]. Of these, 37 studies were lifestyle interventions for children <12 years (four dietary, nine physical activity, twenty-four behavioral). They concluded that it is difficult to recommend any particular intervention, but indicated that family-based lifestyle interventions combining dietary, physical activity and behavioral components can produce ‘a significant and clinically meaningful reduction in overweight’ (p. 2). Parental involvement was identified as useful with children under 12. A review of the limited research on interventions focusing on parenting to treat childhood obesity shows a small to moderate effect on weight-related outcomes [11]; meriting further development. NICE [12] and Oude Luttikhuis *et al*. [10] both point to the paucity of cost-effectiveness studies.

Recent RCTs in the UK on interventions targeting children who are obese have covered a variety of approaches. An RCT of the 9-week family-based community program MEND (Mind, Exercise, Nutrition… Do it!) with 116 children (8 to 12 years) showed a between-group difference in the BMI *z*-score at the 6-month follow-up of −0.24 (95% CI: -0.34 to −0.13, *P* < 0.0001, *n* = 82) in favor of MEND over a waiting list control [13]. A further RCT compared pediatric dieticians using a one-to-one behavioral approach (5 hours) with standard dietetic care (1.5 hours) in 134 children (5 to 11 years) [14]. No significant differences in BMI *z*-scores were found at 6 or 12 months. A feasibility RCT compared the community-based Watch It program (delivered by health trainers) with a waiting list control, in 70 children and adolescents, finding no significant change in BMI *z*-score with treatment [15]. Another RCT has assessed whether Epstein’s ‘family-based behavioral treatment’ is effective in a UK National Health Service (NHS) hospital setting (*n* = 72) [16]. In comparison with a waiting list control, there was no significant difference between the groups in BMI *z*-score, although both treatment and control groups showed significant reductions.

Families for Health is a family-based group intervention for the treatment of children aged 6 to 11 years who are overweight or obese. The program puts greater emphasis on parenting skills, relationship skills and emotional and social development than other similar interventions, and combines this with information about lifestyle. Given the likely effectiveness of parenting interventions in the treatment and prevention of childhood obesity [11], this approach has been investigated as an alternative in the UK. The development and evaluation of the Families for Health program has followed the Medical Research Council (MRC) framework for complex interventions [17]. A pre-post pilot in Coventry of 27 children showed that mean reductions in the children’s BMI *z*-scores from baseline were sustained at 9 months (−0.21, 95% CI: −0.35 to −0.07, *P* = 0.007) [18] and 2 years (−0.23, 95% CI: −0.42 to −0.03, *P* = 0.027) [19]. There were also other health-related improvements. Interview data showed that parents found the parenting approach helpful, providing the tools to become ‘agents of change’ in the family. NHS costs to deliver the program were £517 per family or £402 per child [19].

In this paper we describe the protocol for a randomized controlled trial and parallel economic evaluation of Families for Health and the main implementation challenges we have encountered.

### Aim and objectives

Our aim is to assess the effectiveness and cost-effectiveness at 12 months of the Families for Health program delivered within the NHS using a randomized controlled trial methodology.

Our objectives are to:

•Assess the effectiveness of the Families for Health program in reducing the BMI *z*-score in children aged 6 to 11 years who are overweight or obese.

•Evaluate the cost-effectiveness of the Families for Health program (expressed in terms of incremental cost per quality-adjusted life year gained).

•Investigate parents’ and children’s views of the program and their observations on approaches to maximizing impact.

•Investigate facilitators’ views of the program and their observations on approaches to maximizing impact.

## Methods

### Design

The trial will evaluate the effectiveness of Families for Health in comparison to usual care in children aged 6 to 11 years who are overweight or obese. The design of the trial is a multi-center, randomized parallel group controlled trial with parallel economic evaluation and 12-month follow-up. Participants will be randomized to either of two arms: Families for Health intervention (60 families) or the control group receiving usual care (60 families).

**Figure 1:**
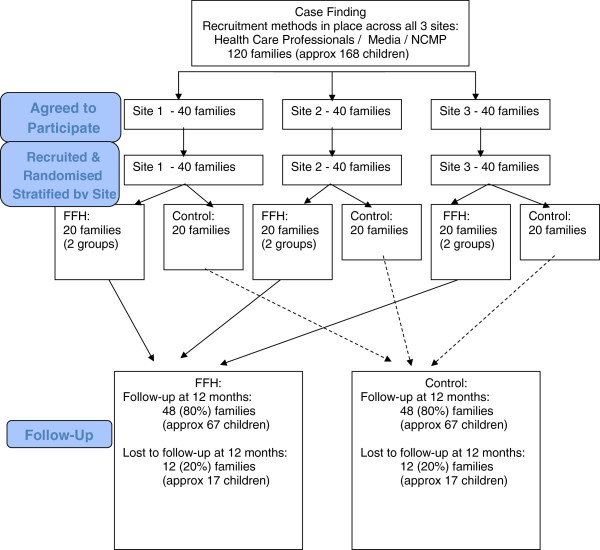
Flow diagram: randomized controlled trial evaluating the effectiveness and cost-effectiveness of Families for Health.

Outcomes will be assessed at baseline, end of program (3 months) and 12 months post randomization, to evaluate both the short-term and sustained effects. A mixed-methods evaluation will run in parallel to the trial. See Figure 1 for the CONSORT flow diagram of families in the trial.

### Setting

The three localities are Primary Care Trusts (PCTs) within the West Midlands: NHS Coventry, NHS Warwickshire and Wolverhampton City PCT.

### Sample size calculation

For power calculations we assumed a residual standard deviation in the BMI *z*-score of 0.22, a standard deviation of the random family effects of 0.14, an intra-cluster correlation of 0.1 in the intervention groups, a two-sided significance of 5% and that 60% of participating families have one overweight or obese child and 40% have two. Allowing for clustering effects by family and for group effects in the intervention arm, a sample size of six groups of ten families (60 families) in the intervention arm and 60 families in the control arm gives a power of 94% to detect an intervention effect of 0.2 in BMI *z*-scores. If 30% of families drop out, the study retains a power of 88%. Power values were estimated using a simulation study including 10,000 simulated trials. Calculations were informed by estimates from the pilot [18]. As a check, we also calculated the power when no families have more than one overweight or obese child. In this case the power is 92% or 83% if 30% of families drop out.

### Recruitment

#### Inclusion criteria

Families must have at least one overweight (≥91st centile for BMI) or obese (≥98th centile for BMI) child aged 6 to 11 years, based on UK 1990 BMI [20].

At least one parent or guardian and the overweight child must be willing to take part.

#### Exclusion criteria

Families where the parent or child has insufficient command of English, who would find it difficult to participate in the group, will be excluded.

Children with a metabolic or other recognized medical cause of obesity will be excluded.

Children with severe learning difficulties and/or severe behavioral problems, who would find it difficult to participate in a group-based program, will be excluded.

The trial will recruit 40 families from each of the three Primary Care Trusts. Each trust will run two Families for Health programs. Families will primarily be recruited via referral from local services, the National Child Measurement Programme (NCMP) or by self-referral following publicity in the local media. Other recruitment methods include school newsletters, distribution of flyers, posters in the local community and recruitment at local health events. Data from the NCMP 2008/9 estimates the pool of potential participants is in excess of 600 children in each of Warwickshire, Coventry and Wolverhampton in Year 6 (10 to 11 years) alone [21].

#### Obtaining informed consent

The researchers will use a three-step procedure to obtain informed consent, giving parents and children time to consider whether they wish to participate. Each potential participant is given or sent by post information sheets about the trial (child and parent versions). After a minimum of 3 days, parents are contacted by telephone to ask whether they are still interested in taking part in the trial and to answer any questions. A researcher then visits the parent(s) and child(ren) at their home, and obtains the parent’s written consent and the child’s written assent. All researchers are trained in informed consent, including methods for assessing competence for consent, agreement to participate and obtaining assent from children.

#### Randomization, allocation concealment and blinding

The random allocation sequence is computer generated and implemented by a central telephone registration and randomization service at the Warwick Clinical Trials Unit. The unit of randomization is the family. Randomization will be stratified by locality (Coventry, Warwickshire, Wolverhampton) using a biased coin (*P* = 2/3) minimization method within each locality to ensure approximately equal numbers of families are randomized to the Families for Health program and control so that viable attendance numbers are obtained for each program (maximum twelve and minimum eight families). Researchers register families after confirming eligibility and obtaining consent, in order to ensure allocation concealment. The families cannot be blinded to treatment allocation. The term ‘control’ is not used at any time with the families and they are assured at the time of consent that they will be receiving one of two possible programs, namely Families for Health or usual care. To date, there is no evidence to suggest that there is more or less attrition from one arm or the other. Every effort is made to ensure that study personnel involved with data collection and analysis remain masked with regard to the data they are collecting or analyzing until the analysis is complete.

### The intervention

Families for Health V2 is a family-based program aimed at the treatment of children (6 to 11 years) who are overweight or obese. It was developed at the University of Warwick by a team including investigators in this trial (WR, SS-B). Following development and evaluation of the original program Families for Health V1 [18,19], changes were made including shortening it from 12 to 10 sessions, adding two follow-up sessions and enhancing the information given to families on healthy eating and pedometers. The program combines information on parenting skills, social and emotional development, as well as lifestyle change. The parenting aspects are based on the Nurturing Programme from Family Links [22], and the circle time elements in the children’s program have parallels with the Family Links Nurturing Programme for schools [23].

Delivery is group based with between 8 and 12 families, with children and parents attending parallel groups. Both parents will be invited, together with all overweight and non-overweight siblings in the target age range. The program is manualized, with detailed handbooks available to facilitators, parents and children. The program is run at a weekend, for 2½ hours each week for 10 weeks. Follow-up sessions are held at 1 and 3 months post-intervention. The intervention is delivered in a community setting (for example, a leisure center or school), to enhance access and ensure adequate space and facilities for physical activity. Families in the intervention arm will be eligible for usual care interventions in addition to attendance at the program. Any usual care they receive will be documented.

Table 1 outlines the main content of the parallel parents’ and children’s groups for the 10 weeks. The topics covered each week are broadly the same for both parents’ and children’s groups to promote greater understanding and discussion at home. The parents’ group covers both support with parenting skills and family lifestyle, which are integrated in the weekly sessions. The approaches include facilitated discussion, role play, goal setting, skill practice, a solution-focused approach and homework. The children’s program includes a focus on healthy eating using the Eatwell plate as the basis; circle time to discuss emotional aspects of their lives and enhancing self-esteem; and physical activity aimed at increasing activity levels by participation in games, the use of pedometers and introduction to new physical activities. The parents and children meet mid-way in each session for a healthy snack and an active game. This gives facilitators an opportunity to act as role models, for example showing parents how they might reward or praise their children, and to introduce ways in which children and parents can interact at home. It also provides an opportunity for children to prepare healthy snacks and to try new foods. Given the age range of the children (6 to 11 years), facilitators of the children’s groups may adapt the delivery of their sessions according to the age of their group, for example, splitting older and younger children during group work, or giving older children added responsibility.

**Table 1:** Content of parents’ and children’s parallel groups for Families for Health (V2)

**Week**	**Parents’ program**	**Children’s program**
1	**Let’s get started**	**Let’s get started**
	What is health?	Why be healthy?
	Balancing act 1: energy in, energy out	Balancing act 1: energy in, energy out
	Let’s look after ourselves	
2	**Balancing acts**	**Balancing acts**
	Discipline (including setting limits and praise)	Balancing act 2: what our bodies need to eat
	Balancing act 2: food our bodies need	The gift of praise
3	**Inner power – our ally for health**	**Inner power – our health helper**
	Family guidelines and rewards	Our inner power
	Finding our power for health (focus on physical activity)	Let’s get active
		Introducing the pedometer
4	**The question of choice**	**Our choices**
	Our eating habits	Making strong choices
	Children’s choices	Let’s go shopping
5	**Health is a family affair**	**Liking ourselves**
	How much we eat (portion sizes)	Glad to be me
	Building self-esteem	Let’s make a rainbow (of fruit & veg)
6	**Feelings – a guide to our emotional health**	**Getting to know our feelings**
	Thinking about feelings	Feeling up, feeling down
	Active alternatives to staring at the screen	Screen savers: what else we can do
7	**Solutions to stress**	**Time to chill out**
	Stress – and what we can do about it	What winds us up
	Coming to our senses	What calms us down
	Surviving at the supermarket	Activity taster
8	**A world of labels**	**Food detectives**
	Food labels: what do they mean?	What’s on the label?
	Labeling our children	Activity taster
9	**Taking charge**	**Living healthily**
	From problem to solution	Problems, puzzles and solutions
	A healthy lifestyle or a life of diets?	Activity taster
	Meeting the challenge of special occasions	
10	**A healthy family future**	(combined session with parents)
	Scaling the ladder to health	
	We are stars!	
	Family party: time to celebrate	

Four facilitators are required to run each program (two each for the children’s and parents’ groups). Facilitators are identified from the local NHS or other services, and selected on the basis of personal attributes including empathy for families with overweight children and previous relevant experience. Professional backgrounds include community nursing, teaching, youth work, leisure services and dietetics. Facilitators attended a 4-day training course provided by Family Links, which covered the content, philosophy and logistics of running the program.

The main principles underpinning the Families for intervention are that parents are identified as the agents of change responsible for implementing lifestyle change in the family [24]. The parenting aspects aim to support and increase parental capacity to implement and maintain the lifestyle changes. The program takes a solution-focused approach with families identifying small changes that they would like to try each week. The focus is on healthy eating (not diet) and activity for the whole family (that is, not just for the child who is overweight), with an emphasis on children growing into their weight rather than weight loss. The program aims to promote a sustainable, healthy approach to family-wide lifestyle change. The Families for Health program differs from the usual care options (described in the following section) in its focus on how the family functions as described above. The program puts greater emphasis on parenting skills, relationship skills and emotional and social development than other similar interventions, and combines this with information about lifestyle. These form a core theme throughout and are integrated alongside topics in each weekly session.

### Usual care control group

Families assigned to the control arm will continue with any support they are receiving and/or will be offered any usual care available in their area. At the present time, usual care for each locality is as follows. Coventry has the One Body One Life program, which is a group-based family intervention [25]. Warwickshire has Change4Life advisors, who offer one-to-one support for weight management for children. In Wolverhampton, usual care is either the WISH (Wolverhampton Inspiring and Supporting Health) weight management program for children and young people aged 7 to 15 years, comprising a two-step program, MEND (Mind, Exercise, Nutrition… Do it!) program and Choose It (focusing on taster sessions for physical activity, healthy eating), or Weight Watchers for young people (accompanied by a parent) aged 10 years and over. Table 2 outlines the main features of the usual care options at all three sites.

**Table 2:** Usual care program details

**Usual care program**	**Delivery**	**Core themes**
One Body, One Life (OBOL) (Coventry)	Parent and child group based	Healthy eating
	1.5 hrs weekly for 10 weeks	Physical activity
	Delivery at school or community venue	Health checks
Change4Life Advisor (Warwickshire)	Parent and child one-to-one with Change4Life advisor	Healthy eating (Eatwell plate, portion sizes, food labeling)
	First session ~1.5 hours, subsequent visits ~45 minutes	Increasing physical activity
	Number of visits varies according to family needs (average five visits)	
	Majority of visits at home, occasionally at school or clinic	
Wolverhampton Inspiring and Supporting Health (WISH) (Wolverhampton)	Parent and child group based	MEND (Mind, Exercise, Nutrition… Do it!) and Choose It (focusing on taster sessions for physical activity and healthy eating)
	2 hrs weekly for 10 weeks	
	Delivery at community venue	
Weight Watchers (10 years plus) (Wolverhampton)	Parent and child group based	Healthy eating (portion sizes)
	1 hr weekly for 12 weeks	Encourage increased physical activity (pedometers for sale)
	Delivery at community venue	

### Outcome measures

The outcome variables will be measured at baseline, at the end of the 10-week Families for Health program (or at approximately 3 months in the usual care arm) and at 12 months post randomization. In families with more than one eligible child who meet the inclusion criteria, data will be collected on all participating children (excluding non-overweight siblings).

### Primary outcome measure

The primary outcome measure is the change in children’s BMI *z*-score at 12 months. Weight will be measured using the Tanita body composition analyzer (model BC-420S MA) to the nearest 0.1 kg, and height measured by a Leicester stadiometer to the nearest 0.1 cm. BMI (kg/m^2^) will be converted into standard deviation scores (*z*) from 1990 UK growth reference curves [20].

### Secondary outcomes

Measurements of the children:

•Change in children’s BMI *z*-score at end of program (3 months from baseline)

•Waist circumference (*z*-score) [26], using a Seca 200 tape

•Percentage body fat with Tanita body composition analyzer

•Time spent in physical activity and intensity, using a 7-day accelerometer recording (ActiGraph GT3x) with step-count function, to give minutes per day spent doing moderate/vigorous physical activity and steps per day. An activity log will be completed in parallel. Evenson’s activity count cut-points for the ActiGraph for physical activity intensities (sedentary, light, moderate, vigorous) will be used for the analysis, as recommended by Trost [27]. This outcome will only be measured at baseline and 12 months in order to minimize seasonal effects [28].

Validated questionnaires completed by the children:

1. Children’s quality of life (PedsQL) [29]

2. Children’s fruit and vegetable consumption using Day in the Life [30]

3. EuroQol EQ-5D-Y health state valuation [31]

Measurement of the parents:

•BMI: height recorded using a Leicester stadiometer and weight with Tanita scales

Validated questionnaires completed by parents:

•Family Eating and Activity Questionnaire: reports of activity of parents and children, and family eating environment [32]

•Children’s quality of life from parents’ perspective (PedsQL) [29]

•Child–Parent Relationship Scale (short form with 15 items) [33]

•Parenting style: Parenting Styles and Dimensions Questionnaire [34]

•EuroQol EQ-5D health state valuation – parent’s own [35]

•EuroQol EQ-5D-Y health state valuation of the child by a parent [31]

•Warwick-Edinburgh Mental Well-Being Scale (WEMWBS) for parents [36]

•Client Services Receipt Inventory (CSRI), recording the services received by each child (adaptation of CSRI from Beecham and Knapp [37])

### Statistical analysis

As indicated above, the primary endpoint for the statistical analysis will be the change in BMI *z*-score after 12 months of follow-up. It is anticipated that these data will be correlated within a family and possibly also within groups in the Families for Health intervention arm. The primary statistical analysis will use hierarchical mixed effect modeling to allow for this multi-level clustering. This will enable unbiased estimation of the effect of the Families for Health intervention together with accurate 95% confidence intervals for this estimate. The model will also adjust for important baseline factors such as baseline BMI *z*-score and gender. Analyses of the secondary endpoints listed above will also be conducted using hierarchical models or marginal modeling in a similar way to that for the primary endpoint, possibly after transformation to ensure approximate normality. Analysis will be conducted using R, SAS or MLwiN as considered most appropriate. All main analyses will be conducted on an intention-to-treat basis, comparing the groups as randomized irrespective of whether they attended all or any of the Families for Health intervention or of the intervention(s) received as part of usual care.

The primary analysis will be conducted at the conventional (two-sided) 5% level. It is not proposed to formally adjust for multiple testing amongst the secondary endpoints as these are likely to be highly correlated so that adjustment techniques such as the Bonferroni method are likely to be conservative. The number of analyses conducted will, however, be informally considered when interpreting the results of these statistical analyses.

As the support offered as usual care may vary, a secondary analysis will seek to compare the effect of the intervention actually received by each family.

A fully specified data analysis plan will be developed in the early phases of the trial and approved by the Trial Steering Committee. Data will be summarized and reported in accordance with CONSORT guidelines for randomized controlled trials [38]. No interim analyses are anticipated. Safety data will be monitored by the Data Monitoring and Ethics Committee.

### Economic evaluation

The economic evaluation will be conducted from the recommended NHS and Personal Social Services perspectives [39]. Data will be collected on all significant resource inputs used in the care of each family during the period between randomization and one year post-randomization using a childhood adaptation of the Client Services Receipt Inventory [37]. A particular focus of the economic evaluation will be a full assessment of the cost of delivering the intervention in community settings, including the costs of training and developing facilitators, staff-related expenses, and revenue and capital overheads. The unit costs of each resource item will be valued using both primary research, based on established accounting methods, and data collated from secondary national tariff sets.

The results of the baseline economic evaluation will be expressed in terms of incremental cost per quality-adjusted life year (QALY) gained. QALY data will be estimated using responses to the EQ-5D-Y (EQ-5D youth version); evidence of its feasibility, reliability and validity have been reported in the literature [31]. Both the parents and children will be asked to independently complete the EQ-5D-Y at each time point of assessment. Each parent will be asked to complete the EQ-5D-Y as he/she would expect the child to respond (as opposed to asking the parent to rate the child’s health from his/her perspective). We shall use non-parametric bootstrap estimation to derive 95% confidence intervals for mean cost differences between the trial groups and to calculate 95% confidence intervals for incremental cost effectiveness ratios [40]. A series of sensitivity analyses will be undertaken to explore the implications of uncertainty on the incremental cost-effectiveness ratios and to consider the broader issue of generalizability of the study results. This will include a sensitivity analysis that assesses the potential impact of adopting a societal perspective for the economic evaluation using data on informal and indirect costs provided by parents in the economic questionnaires. In addition, cost-effectiveness acceptability curves will be constructed using the net benefits approach [41].

A *de novo* decision-analytic model will also be developed in order to estimate the lifetime cost-effectiveness of the Families for Health intervention. The model will be informed both by data collated by the family-completed economic questionnaires, but also by data extracted from secondary sources. The model will be structured using published evidence on the epidemiology and natural history of childhood obesity, and will be informed by guidance from clinical advisors, to reflect the natural course of childhood obesity and the impact of alternative interventions. Accepted guidelines for good practice in decision-analytic modeling and the general principles outlined in the NICE reference case will be followed [39,42]. In developing and populating the model, three issues will be considered of central importance to the approaches and methods employed: (i) the need to develop an evidence network that facilitates direct and indirect comparisons between interventions; (ii) the requirement to extrapolate outcomes beyond the time horizon of the main RCT, to ensure that differences in costs and QALYs are appropriately quantified; and (iii) the need to ensure that the data inputs and assumptions are relevant for informing current NHS practice. Long-term costs and health consequences will be discounted to present values using discount rates recommended for health technology appraisal in the United Kingdom [39].

### Process evaluation

Issues to be addressed by the process evaluation are:

•What is the best method of recruiting families?

•Which, if any, aspects of a healthier lifestyle do the Families for Health and usual care programs enable? Which of these are sustainable to the 12-month follow-up?

•What are the families’ (parents and children) experiences of Families for Health and the usual care programs, and how might they be improved?

We will assess the delivery of the program and how it is received by families as implementation takes place, using the framework for process evaluation developed by Linnan and Steckler [43], see Table 3. An important aspect of process evaluation is to assess the level of difficulty of recruiting participating families and the effectiveness of the various methods of recruitment. Active recruitment methods, such as referral by a doctor or targeted mail shots to families with children who are obese, have been shown to be more effective than passive recruitment (for example, articles in the media) in some circumstances in enrolling families into an RCT, although passive recruitment methods were better at retaining families from enrollment to randomization [44]. In the pilot of this program we found self-referral following articles in the local media to be the most successful recruitment strategy, giving higher completion rates than recruitment via health professionals [18]. We will assess the effectiveness of these two approaches together with a third approach of targeted recruitment via the NCMP, which has measured children in Reception (4 to 5 years) and Year 6 (10 to 11 years) in state schools in England since 2005 to assess the prevalence of overweight and obesity [45].

**Table 3:** Framework for process evaluation

**Component**	**Definition**	**How assessed in evaluation**
Recruitment	Success of methods used to approach and recruit participants	Baseline questionnaire asking parents how they heard about the trial and how they were referred
Reach	Degree to which an intended audience participates in an intervention	Baseline questionnaire with parents asked about socio-demographic characteristics, to define if participants reflect the locality populations and if any sub-groups were more or less likely to participate
Dose delivered	The amount of intervention provided by the intervention team	(1) Facilitators will keep notes about unavoidable changes to the program enabling assessment of the number of sessions delivered as planned
		(2) Facilitators’ weekly evaluation forms
		(3) Recording of additional interventions or care accessed by both groups
Dose received	Extent of engagement with the intervention by the target population	(1) Facilitators will log attendance by families, including withdrawals
		(2) Parents’ weekly evaluation questionnaires of the sessions
		(3) Parents end-of-program questionnaire and interviews, reporting changes made
Fidelity	The extent to which the intervention was delivered as planned, that is, the quality and integrity of the intervention	(1) For three to four sessions (randomly selected) on each Families for Health program, the fidelity will be addressed indirectly from:
		- flip-charts used and developed during the session
		- the parents’ end-of-session evaluation, covering whether the session’s topics were mentioned and their perception of the facilitators and the program
		- facilitators’ weekly log of their delivery of the program, recording how it went and any variations
		(2) Interviews with facilitators at the end of the program
		(3) Parents’ end-of-program questionnaire

### Interview study

We will undertake one-to-one interviews at the end of the program and at 12 months post randomization with up to 24 children and 24 parents from both arms (Families for Health and usual care). Purposive sampling will include representation from all Families for Health groups and the various usual care options, and will aim for diversity of age and gender of the children, family size and whether they completed the intervention or not. We will aim to interview at least one parent of each interviewed child so we can triangulate the data. Children from families with two or more children in the study will be interviewed together. The interviews will capture participants’ views of the research and of the program, the changes they have made (or otherwise), the facilitating or inhibiting factors they experienced and, where relevant, participants’ reasons for dropping out of the interventions. Interviews at 12 months will include whether any changes made have been sustained or not. Children’s interviews will include draw-and-write techniques as well as a discussion about the program.

The facilitators of each Families for Health intervention will be interviewed at the end of the program as a group. This will explore the aspects of the program they felt worked or did not work, the group dynamics, and an exploration of the main enablers and barriers to change in the families. Finally they will be asked to consider whether they felt the training equipped them to deliver the program.

All interviews will be digitally recorded and transcribed. Analysis of initial interviews will inform development of the interview schedule for further interviews. NVivo software will be used for data handling and coding. Coding will be thematic [46], based on the interview schedule with the addition of emergent themes. After initial coding the qualitative team will meet to review the interviews and coding and discuss further analysis. The quantitative data collected from each of the parent/child interviewees will provide background data for the analysis, with triangulation of qualitative and quantitative data sources. In addition to thematic analysis of all interviews, where there are interviews with a parent and child, these interviews will be linked and comparative analysis undertaken to identify contrasts and similarities in what they report.

### Ethics and research governance

Ethical approval for the study was obtained from the NRES Committee West Midlands - Coventry & Warwickshire, REC (Reference 11/WM/0290) on 03 October 2011. Four amendments were subsequently approved: (1) The Client Services Receipt Inventory was developed and given ethical approval prior to piloting (Substantial Amendment 1, 17 January 2012). (2) The inclusion criterion was changed from children aged 7 to 11 years to 6 to 11 years (Amendment 2, 27 April 2012). (3) The formatting of the CSRI was changed after piloting, the poster for recruitment was revised and the letter used for recruitment via the NCMP was changed (Amendment 3, 21 May 2012). (4) Interviews were originally going to be with just the families who attended the Families for Health program, but the protocol was changed to include interviews with usual care families as well (Substantial Amendment 4, 6 August 2012).

The trial is sponsored by the University of Warwick. NHS Research and Development approvals have been obtained with participating NHS Trusts. A Trial Steering Committee and Data Monitoring and Ethics Committee have been convened. These committees will advise on changes to the protocol, ethical issues and oversee the management of the trial.

The trial is being conducted in accordance with the Standard Operating Procedures of the Warwick Clinical Trials Unit. All data will be stored securely and anonymized in accordance with the Data Protection Act, and the trial will be conducted in compliance with the principles of MRC Good Clinical Practice (GCP) guidelines, the Declaration of Helsinki and other requirements as appropriate.

## Discussion

This paper has presented the protocol for the randomized controlled trial of the Families for Health program. The trial has completed its set-up phase and started recruiting families from March 2012. In this section we will firstly discuss the changes made to the original protocol and then the challenges to implementation.

Two changes have been made to the original protocol. Firstly, two months after recruitment had started, the inclusion criterion for children was changed from being aged between 7 and 11 to being between 6 and 11. Our original recruitment strategy was based on the age range in junior schools; it was felt that extending the age range down to 6 years might alienate older children and prevent provision of a safe space for the 6 year olds. However, during the early stages of recruitment a number of parents of 6-year-old children contacted the trial team with an interest in taking part. These children were eligible for usual care and were keen to take part in the trial. At the same time the parents of some 11-year-old children reported that their children were focusing on the transition to secondary school and did not want to start a primary school age program. Families for Health V2 with its focus on active games, the activities around healthy eating and circle time, suits younger children very well, and including 6-year-old children compensated for the low levels of recruitment from 11 year olds.

During the time period between submission of the grant application and the award of the grant and start-up of the trial, the provision of usual care in the trial localities changed. By the time the trial started all three localities had started to provide evidence-based interventions for overweight and obese children. It was felt to be important to capture parents’ views on usual care as well as their views of Families for Health, so a further protocol amendment was requested to enable interviews with parents and children allocated to usual care, to compare the experience of families in the two arms.

Challenges to the implementation of the study included securing excess treatment costs to fund the delivery of the intervention, the recruitment of families, changes to usual care and introducing strategies to maintain blinding of researchers.

Excess treatment costs are ‘the patient care costs which would continue to be incurred (by the NHS) if the patient care service in question continued to be provided after the R&D study had stopped’, and are the responsibility of the NHS [47]. At a time of a major NHS reorganization in which public health services are transferring from the NHS to local authorities, securing the excess treatment costs to cover the running of the Families for Health intervention (facilitators, venues and consumables) at the three localities was complex. As there was uncertainty about the budget to meet excess treatment costs, these costs were identified by the Public Health Departments from their 2011/12 budget whilst they were still located in NHS Coventry and NHS Warwickshire Primary Care Trusts, and transferred to the University of Warwick in March 2012. Wolverhampton identified funds for groups running in 2012/13 but reorganization may prove an issue for funding groups in 2013/14. As the excess treatment costs were transferred by two sites, this means the University of Warwick has to employ the facilitators of the Families for Health intervention for both Coventry and Warwickshire. This introduced additional complexity for the set-up of the research including obtaining research passports.

The NHS reorganization has had a further effect on the trial because many of the staff in the employ of the PCTs, who are either supporting the trial or who have been trained as facilitators, are experiencing stress relating to the reorganization, for example, due to actual or threatened redundancy. This has meant that the pool of trained facilitators is declining and finding facilitators to run groups is more challenging. It has also meant that those supporting the delivery of the trial within PCTs, who have offered unflagging help to date, inevitably have other major pressures on their time and cannot offer the same level of support.

However, the main challenge of the trial relates to recruitment. Families for Health V2 is a closed group intervention (that is, families start the intervention together), and as such it is necessary to have a group of 8 to 12 families randomized to that arm in order for the intervention to run. As the program involves school age children, takes 10 weeks to run and tends to lose families if there is a significant break in provision, it is only practical to start the program three times a year – at the beginning of the school terms. As approximately half the families will be randomized to usual care, it is necessary to recruit around 20 families willing to be randomized in any locality in order for the intervention to run. This has been challenging and may mean that some groups run with a sub-optimal number of families. For example, the first group in Coventry ran in May 2012 with eight families. Running a group at reduced capacity may impact on effectiveness, as reported in the WATCH IT feasibility RCT [15], and will impact on cost effectiveness.

The National Child Measurement Programme is relatively new and has been developed in different localities in a variety of ways. The planned recruitment via the NCMP was problematic as recruitment to the trial started in March 2012 when most of the PCTs had completed their annual NCMP measurements for the year. Coventry PCT re-sent a letter to a selected group of families advising them of the research study, and this was helpful in recruiting families. Future Families for Health programs are planned to coincide with the timing of the NCMP in each locality. Additional recruitment methods have also been added to the protocol including leaflet drops, attendance at health events where offers of height and weight measurements are made to families, and paid advertisements.

Another key issue is changes to usual care. When the protocol was submitted for funding (January 2010) very few options were open to support families with childhood obesity. However, much has changed over the last 2 to 3 years as local services strive to meet new policy targets and objectives and invest significantly in the development and evaluation of programs. These changes mean that Families for Health V2 is now being assessed against a variety of possible approaches to supporting families. The usual care that children and their families in the control arm receive will be carefully documented. If significant numbers of control families take up usual care offers, our trial may be underpowered because the estimated difference in BMI *z*-scores between control and intervention groups is likely to be reduced.

A further challenge is to maintain the blinding of investigators who are taking the research measurements to the families’ allocations. The nature of the 3-month visit unblinds the researcher as both the responses to the CSRI [37] and the interview are likely to reveal the group allocation. Strategies in place to maintain blinding include letters sent in advance to the parents to ask them not to reveal their allocation to the researchers until required. During the 3-month follow-up visit, researchers take all anthropometric measurements first and the other questionnaires are then completed, prior to the CSRI and then the interview (if required). To date, researchers who are involved in data collection at the 3-month follow-up have successfully remained blinded to the allocation group until the CSRI was administered in all but one family. The researcher who carries out the 3-month follow-up subsequently becomes unblinded so that a different researcher will carry out the 12-month follow-up with this family. The trial administrator will keep a record of which researcher is unblinded to which families, and follow-up visits will be set up accordingly.

### Trial status

This is an ongoing trial (62 of 120 families recruited).

## Abbreviations

BMI: Body mass index; CONSORT: Consolidated Standards Of Reporting Trials; CSRI: Client Service Receipt Inventory; FFH: Families for Health; GCP: Good Clinical Practice; HTA: Health Technology Assessment; MEND: Mind, Exercise, Nutrition… Do it!; MRC: Medical Research Council; NCMP: National Child Measurement Programme; NHS: National Health Service; NICE: National Institute for Health and Clinical Excellence; NIHR: National Institute for Health Research; PedsQL: Pediatric quality of life inventory; PCT: Primary Care Trust; QALY: Quality-adjusted life year; RCT: Randomized controlled trial; SAS: Statistical Analysis System; UK: United Kingdom; WEMWBS: Warwick-Edinburgh Mental Well-Being Scale

## Competing interests

The authors declare that they have no competing interests.

## Authors’ contributions

SS-B and WR were involved in the conception, design and fund-raising for the research and development of the Families for Health program. SS-B, WR, SP, NS, MT, RL, FG and DS designed this trial, were co-applicants on the grant application and are involved in its implementation. JK, KK, TH, GR, KR and FP are involved in its implementation. All authors have been actively involved in the authorship of the paper, and all approved the final manuscript.
